# Clozapine protects adult neural stem cells from ketamine-induced cell death in correlation with decreased apoptosis and autophagy

**DOI:** 10.1042/BSR20193156

**Published:** 2020-01-24

**Authors:** Mathias Lundberg, Sophie Curbo, Hannes Bohman, Ingrid Agartz, Sven-Ove Ögren, Cesare Patrone, Shiva Mansouri

**Affiliations:** 1 Department of Clinical Science and Education, Södersjukhuset, Internal Medicine, Karolinska Institutet, Stockholm, Sweden; 2 Centre for Psychiatry Research, Department of Clinical Neuroscience, Karolinska Institutet, Stockholm, Sweden; 3 Department of Laboratory Medicine, Division of Clinical Microbiology, Karolinska Institutet, Stockholm, Sweden; 4 NORMENT, KG Jebsen Centre for Psychosis Research, Division of Mental Health and Addiction, Institute of Clinical Medicine, University of Oslo, Oslo, Norway; 5 Department of Psychiatric Research, Diakonhjemmet Hospital, Oslo, Norway; 6 Department of Neuroscience, Karolinska Institutet, Stockholm, Sweden

**Keywords:** autophagy, clozapine, fluoxetine, haloperidol, ketamine, neuroprotection

## Abstract

Adult neurogenesis, the production of newborn neurons from neural stem cells (NSCs) has been suggested to be decreased in patients with schizophrenia. A similar finding was observed in an animal model of schizophrenia, as indicated by decreased bromodeoxyuridine (BrdU) labelling cells in response to a non-competitive N-methyl-d-aspartate (NMDA) receptor antagonist. The antipsychotic drug clozapine was shown to counteract the observed decrease in BrdU-labelled cells in hippocampal dentate gyrus (DG). However, phenotypic determination by immunohistochemistry analysis could not reveal whether BrdU-positive cells were indeed NSCs. Using a previously established cell model for analysing NSC protection *in vitro*, we investigated a protective effect of clozapine on NSCs.

Primary NSCs were isolated from the mouse subventricular zone (SVZ), we show that clozapine had a NSC protective activity alone, as evident by employing an ATP cell viability assay. In contrast, haloperidol did not show any NSC protective properties. Subsequently, cells were exposed to the non-competitive NMDA-receptor antagonist ketamine. Clozapine, but not haloperidol, had a NSC protective/anti-apoptotic activity against ketamine-induced cytotoxicity. The observed NSC protective activity of clozapine was associated with increased expression of the anti-apoptotic marker Bcl-2, decreased expression of the pro-apoptotic cleaved form of caspase-3 and associated with decreased expression of the autophagosome marker 1A/1B-light chain 3 (LC3-II).

Collectively, our findings suggest that clozapine may have a protective/anti-apoptotic effect on NSCs, supporting previous *in vivo* observations, indicating a neurogenesis-promoting activity for clozapine. If the data are further confirmed *in vivo*, the results may encourage an expanded use of clozapine to restore impaired neurogenesis in schizophrenia.

## Introduction

Schizophrenia is a devastating mental disorder characterised by severe cognitive impairments, involving severe deterioration of executive function, attention and memory [1]. Moreover, a body of evidence indicates that patients with schizophrenia have disturbed neural plasticity, generally defined as a process responsible for the ability of the brain to adapt to new environmental changes and/or recover from injuries by restructuring itself [2,3]. Neural plasticity has recently been shown to be dependent on adult neurogenesis [4].

Neurogenesis, i.e. the production of new neurons from neural stem cells (NSCs), has been shown to be a continuing process throughout life in the subgranular zone (SGZ) of the hippocampal dentate gyrus (DG) and in the subventricular zone (SVZ) of the lateral ventricle. The vast majority of cells from the SVZ provide cellular turnover in the olfactory bulb (OB) in the rodent brain [5], and in the striatum in the human brain [6]. The SGZ provides cells to the hippocampus in both humans and rodents [7,8]. Impaired neurogenesis and/or insufficient regenerative activity, has been observed in various CNS diseases/disorders, including Alzheimer’s disease and schizophrenia [9–11], as well as in a number of experimental animal models [12,13]. Whether this impairment is causative and/or the result of the CNS disorders themselves is unknown. However, studies indicate that impaired functional neurogenesis may be restored by the use of neuroprotective agents [14,15]. This may be in accordance with current schizophrenia literature, where neuroprotection has been suggested to be a novel drug target for the treatment of this disease [16].

Due to the complexity to study the role of neurogenesis in human schizophrenia, several animal models have been designed. Administration of a non-competitive N-methyl-d-aspartate (NMDA) receptor antagonist, ketamine and phencyclidine (PCP) [17,18] are commonly used to mimic symptoms in rodents similar to those associated with schizophrenia in humans [17]. Moreover, animals exposed to ketamine acutely and repeatedly, have shown to develop behavior changes and brain morphological changes similar to those observed in schizophrenic patients [17,19]. We and others have shown that ketamine has dose-dependent cytotoxic activity on numerous cell types, including adult NSCs. The associated toxicity correlated with endoplasmic reticulum stress, mTOR activity, mitochondrial dysfunction and apoptosis [20–26]. Moreover, in the ketamine animal model of schizophrenia, neurogenesis has been shown to be activated, but without the capacity to overcome the simultaneous ketamine-mediated loss of parvalbumin expressing neurons, indicating a deficiency and/or impairment in neurogenesis [12].

Antipsychotics and antidepressants (as adjunctive agents), are commonly used in the treatment of schizophrenia [27–29]. Antidepressants have been shown to promote neurogenesis in hippocampus and SVZ [30,31] by promoting NSC proliferation. Antidepressants have also been shown to promote cell protection on hippocampus-derived NSCs [32]. Antipsychotics, the mainstay of treatment for schizophrenia, are commonly divided into two classes: the first-generation (FGA), including haloperidol and the second-generation (SGA) including risperidone, olanzapine and clozapine [29]. It is well-established that SGA are more potent than FGA in treating symptoms of schizophrenia. The reason for the difference has not been clarified. However, recent studies indicate that they may differ in their capacity to promote neurogenesis [28,33].

Clozapine, the most effective drug for treatment-resistant schizophrenia [34], has been shown to stimulate cell proliferation in the hippocampus, as indicated by increased labelling of bromodeoxyuridine (BrdU), suggesting a neurogenic-promoting activity for clozapine [35,36]. However, phenotypic analysis by immunohistochemistry staining of BrdU-positive cells could not clarify that the proliferating cells were indeed NSCs [35]. Moreover, in an animal model of schizophrenia induced by the NMDA-receptor antagonist PCP, clozapine was shown to normalise/counteract a decrease in neurogenesis in DG [37], indicating that clozapine has a NSC-protective activity [34]. However, phenotypic analysis by immunohistochemistry could not reveal that the protective activity of clozapine on BrdU-positive cells were indeed NSCs. In the same study, clozapine alone could not be shown to promote an increased number of BrdU-positive cells, as previously observed.

Phenotypic analysis by immunohistochemistry to detect NSCs *in vivo* has been shown to be difficult due to the deficiency of reliable NSCs markers, thus making cellular *in vitro* cell models essential.

*In vitro* studies have shown that clozapine may also have cell protective properties in various cells types including microglia cells and rat pheochromocytoma cells (PC12) [38–40]. However, it is not known whether clozapine also has NSC protective properties, involving up-regulation of an anti-apoptotic response *in vitro*. Haloperidol, the most well-representated FGA drug used worldwide has shown opposing activity to clozapine. In contrast with clozapine, haloperidol has shown neurotoxic activity both *in vitro* and *in vivo* and no obvious effect on neurogenesis [35–37].

In the present study, we have tested the hypothesis that clozapine has NSC protective activity, involving up-regulation of an anti-apoptotic response on adult NSCs. We show that clozapine had NSC-protective activity alone and against ketamine-induced cytotoxicity *in vitro*. Moreover, this activity of clozapine was associated with attenuated expression of apoptosis markers and decreased expression of the autophagosome marker 1A/1B-light chain 3 (LC3-II). In summary, our findings indicate that clozapine may play a protective/anti-apoptotic role on NSCs, supporting previous *in vivo* observations of neurogenic-promoting activity for clozapine. If these data are confirmed *in vivo*, the results may encourage an expanded use of clozapine to restore impaired neurogenesis in schizophrenia.

## Materials and methods

### NSCs isolation and cell cultures

The SVZ of the lateral brain ventricles of adult male mice 6 weeks of age (five C57 BL6/SCA mice in each experiment) was micro-dissected by using a micro-dissector scissor and enzymatically dissociated in 0.5 mg/ml trypsin, 0.8 mg/ml hyaluronidase and 80 U/ml deoxyribonuclease I (Sigma–Aldrich, St. Louis, MO) in DMEM/F12 containing B27 supplement, 4.5 mg/ml glucose, 100 U/ml penicillin, 100 μg/ml streptomycin sulphate and 12.5 mM HEPES buffer solution (Invitrogen, Stockholm, Sweden). The enzymatic digestion was carried out at 37°C for 20 min. After a gentle trituration with a pipette and mixing, cells were passed through a 70-μm strainer (BD Biosciences, Stockholm, Sweden) and pelleted at 1000 rpm for 12 min. The centrifugation step was repeated once more after removing the supernatant by adding fresh cold DMEM/F12. The supernatant was then removed, and cells were re-suspended in DMEM/F12 supplemented with B27 and 18 ng/ml human epidermal growth factor (EGF) (R&D Systems, Oxon, U.K.). Cells were plated in a 10-cm Petri dish and incubated at 37°C for 7 days in order for neurospheres (NSs) to be developed. After 7 days, the NSs were collected and centrifuged at 1000 rpm for 10 min. NSs were re-suspended in 0.5% trypsin/EDTA (Invitrogen, Stockholm, Sweden), by incubating at 37°C for 2 min and triturated gently to aid dissociation. After a further 2-min incubation at 37°C, the cell preparation was diluted 1/20 in DMEM/F12 at 37°C. Cells were then pelleted at 1000 rpm for 10 min and re-suspended in fresh DMEM/F12 containing 18 ng/ml EGF and 16 ng/ml human basic fibroblast growth factor (bFGF) (R&D Systems, Oxon, U.K.) before plating. NSCs were split every 5 days for 4 weeks and all experiments were performed between passages 2 and 8. The NSC culture has been characterised and validated in a previous work by Mercer et al. [41].

All experiments were conducted according to the regional ethics committee for animal experimentation conforming to the ‘Guide for the Care and Use of Laboratory Animals’ published by U.S. National Institutes of Health (NIH publication # 80-23, revised 1996). The C57 BL6/J mice were imported from Nova-SCB, Stockholm, Sweden. The regional ethical committee (Stockholm Södra djurförsöksetiska nämnd, ethical permit number N422/12 and 423/12) approved all animal studies presented in the manuscript. Animals were killed in a CO_2_ chamber by cervical dislocation at the Animal Department, Karolinska Institutet, Stockholm, Sweden.

### Ketamine medium

Previous work by our group have demonstrated a cytotoxic effect of ketamine on NSCs [24]. NSCs were treated with s-ketamine, NMDA receptor antagonist, which in mice have shown to induce hippocampal atrophy and pathology in parvalbumin-expressing interneurons, and cognitive deficits in a similar way to those observed in schizophrenic patients [17,19]. The indicated ketamine concentration (see ‘Results’ section) was added to DMEM/F12 and supplemented with B27 and 0.01 ng/ml of EGF.

### ATP assay

Previous studies have demonstrated that intracellular ATP levels correlate to cell numbers [42]. In order to measure NSC viability, NSCs were plated as single cells (see above) into 96-well plates (Corning B.V. Life Sciences, Amsterdam, Netherlands) at a final concentration of 50000 cells/well with DMEM supplemented with B27 and low EGF (0.01 ng/ml). To note, the supplementation of low EGF concentration has been adjusted to a minimum level, to produce an experimental condition, where no proliferation is occurring (including the control cells, clozapine, fluoxetine and haloperidol alone and treated cells). The rational for this has been to produce a cellular protection assay in absence of proliferation. Clozapine (1, 10, 100, 500 nM, 2 μM), fluoxetine (1, 10, 100 nM) and haloperidol (250, 500 nM, 1 μM) were co-treated with or without ketamine. After 24 h of incubation at 37°C (5% CO_2_, 98% humidity), intracellular ATP levels were measured using the Cellular ATP Kit HTS according to the manufacturer’s instructions (BioThema, Stockholm, Sweden). In these experiments, the effect of each treatment at a certain concentration was determined in quadruplicates in six to seven different sets of experiments.

### Western blotting

NSCs were plated as single cells and expanded in a 10-cm Petri dish with EGF/bFGF (see under cell cultures). After 3–4 days when NSs were formed, the different treatments were added for 24 h with low EGF concentration (0.01 ng/ml). After exposure, cells were washed with PBS and lysed in a buffer containing 150 mM NaCl, 20 mM Tris, 0.1% SDS, 1% Triton X-100, 0.25% Na-deoxycholate, 1 mM Na_3_VO_4_, 50 mM NaF, 2 mM EDTA, and Protease inhibitory cocktail (Sigma–Aldrich) on ice for 30 min. Samples were clarified by centrifugation. The supernatants were transferred to new tubes and the total protein concentration was determined by Lowry protein assay (Bio-Rad Laboratories, Stockholm, Sweden). Samples were then mixed with reducing SDS/PAGE sample buffer and boiled for 5 min before performing SDS/PAGE. After electrophoresis, proteins were transferred on to polyvinylidene fluoride (PVDF) membranes (Bio-Rad Laboratories). Immunoblot analyses were performed with antibodies against the cleaved form of caspase-3 (1:1000, polyclonal), LC3-II (1:1000, polyclonal) (Cell Signaling Technology, Danvers, MA, U.S.A.), and Bcl-2 (1:1000, monoclonal rabbit) (Abcam, Cambridge, MA, U.S.A.). Immuno-reactive bands were developed using ECL (GE Healthcare, Stockholm, Sweden), imaged with a GelDoc system and quantified with Quantity One software (Bio-Rad Laboratories). After imaging, in order to verify equal protein loading, the PDVF membranes were stained with β-actin (1:800, polyclonal) (Santa Cruz Biotechnology. Inc, Germany) or Coomassie Blue (Fermentas, St. Leon-Rot, Germany) as demonstrated in previous studies [24,43–45]. In these experiments, the effect of each treatment at a certain concentration was determined in single/double samples in three to five different sets of experiments.

### Mitochondrial DNA analysis

NSCs were plated as single cells and expanded in a 10-cm Petri dish with EGF/bFGF (see under cell cultures) for 3–4 days. When NSs were formed, the different treatments were added for 24 h with low EGF concentration (0.01 ng/ml). After exposure, the number of mitochondrial DNA (mtDNA) copies per diploid nucleus in cells were purified and determined by real-time PCR absolute quantification using the ABI 7500 Fast system (Applied Biosystems). Total genomic DNA was purified from mouse stem cells using the DNeasy blood and tissue kit (Qiagen). A total of 10 ng of genomic DNA was used in each reaction. Primers and probe for mouse *mt-ND1* gene (mitochondrial encoded NADH dehydrogenase 1; primers, mt-ND1-F: 5′-TCG ACC TGA CAG AAG GAG AAT CA-3′ and mt-ND1-R: 5′-GGG CCG GCT GCG TAT T-3′; probe, mt-ND1: FAM-AATTAGTATCAGGGTTTAACG-TAMRA) and for single-copy mouse *RPPH1* gene (nuclear-encoded ribonuclease P RNA component H1; primers, RPPH1-F: 5′-GGA GAG TAG TCT GAA TTG GGT TAT GAG-3′ and RPPH1-R: 5′-CAG CAG TGC GAG TTC AAT GG-3′; probe, RPPH1: FAM-CCGGGAGGTGCCTC-TAMRA) were used. For each DNA sample, the mitochondrial gene *mt-ND1* and the nuclear gene *RPPH1* were quantified separately. Standard curves were generated using known numbers of a plasmid containing one copy of each of the two mouse genes. According to the standard curve, the number of copies from each gene was calculated for each sample, and the number of mtDNA copies per diploid nucleus was calculated according to the formula: mtDNA copies per diploid nucleus = 2 × (*mt-ND1* gene copies/*RPPH1* gene copies). In these experiments, the effect of each treatment at a certain concentration was determined in single samples in three to five different sets of experiments.

### Statistical analysis

The differences between groups were tested with one-way ANOVA followed by post hoc Fisher LSD test or Kruskal–Wallis followed by Dunn’s test if data were not normally distributed. All statistical analyses were performed using Sigma Plot software v. 11. Data are presented as mean ± SEM. *P*<0.05 was considered statistically significant.

## Results

### Clozapine and fluoxetine, but not haloperidol increases NSC viability

**Figure 1:**
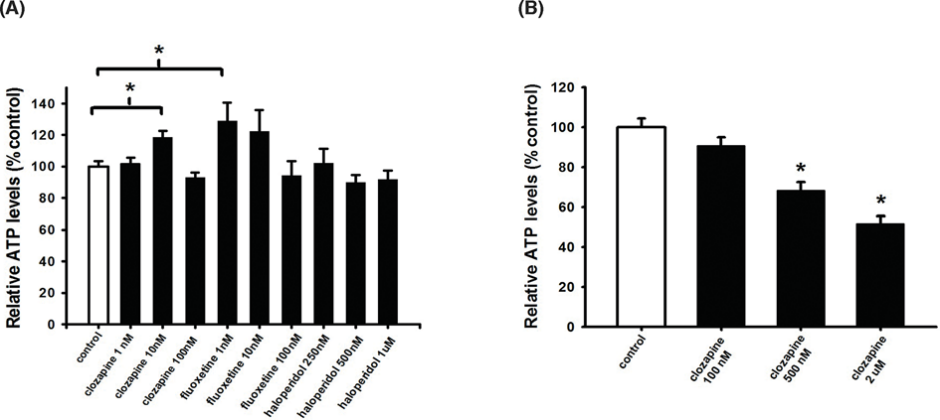
Low clozapine and fluoxetine concentrations increase NSC viability, while haloperidol did not NSCs were plated as single cells treated with (**A**) 1, 10, 100 nM of clozapine or 1, 10, 100 nM of fluoxetine or 250, 500 nM, 1 μM of haloperidol or (**B**) 100, 500 nM, 2 μM clozapine. To measure cell viability, intracellular ATP levels were measured after 24 h. Data are shown as mean ± SEM ((A), *n*=22–35; (B), *n*=15–29). Fisher LSD test and Kruskal–Wallis followed by Dunn’s test was used. Differences were considered significant at *P*<0.05. * denotes *P*<0.05 compared with control.

To determine the potential cell protective activity of clozapine on NSCs, NSCs were isolated from the SVZ and exposed to the indicated concentrations of clozapine. For comparison, we also tested the FGA drug haloperidol and the antidepressant drug fluoxetine, drugs commonly used in the treatment of schizophrenia. NSC viability was assessed after 24 h by measuring intracellular ATP levels. The results in Figure 1A show that both clozapine and fluoxetine significantly increased NSC viability at doses of 10 nM (clozapine) and 1 nM (fluoxetine), respectively, while none of the haloperidol concentrations increased NSC viability. At higher concentrations of clozapine, 500 nM and 2 μM, respectively, NSCs viability was significantly decreased (Figure 1B).

### Clozapine counteracts ketamine-induced decrease in NSC viability

Ketamine administration is a well-established approach to experimentally mimic some aspects of schizophrenia [17]. Previous work by us has shown that ketamine has a dose-dependent cytotoxic activity on mouse adult NSCs after 24-h treatment. Our previous work indicated that 400 μM ketamine induced a significant and submaximal negative effect on NSC cell viability [24]. This cytotoxic concentration is in accordance with several animal and human clinical studies [19,46–49]. In the present study we showed, with this specific concentration, that the copy-number of mtDNA increased significantly after 24-h treatment (data not shown), a sensitive index indicating cellular oxidative stress, inflammation and mitochondrial dysfunction [50,51]. Our result is in accordance with previous work showing an association between an altered mtDNA copy-number and cellular stress/schizophrenia pathology [52–54]. We found that treatment for 24 h significantly increased the copy number of mtDNA.

**Figure 2:**
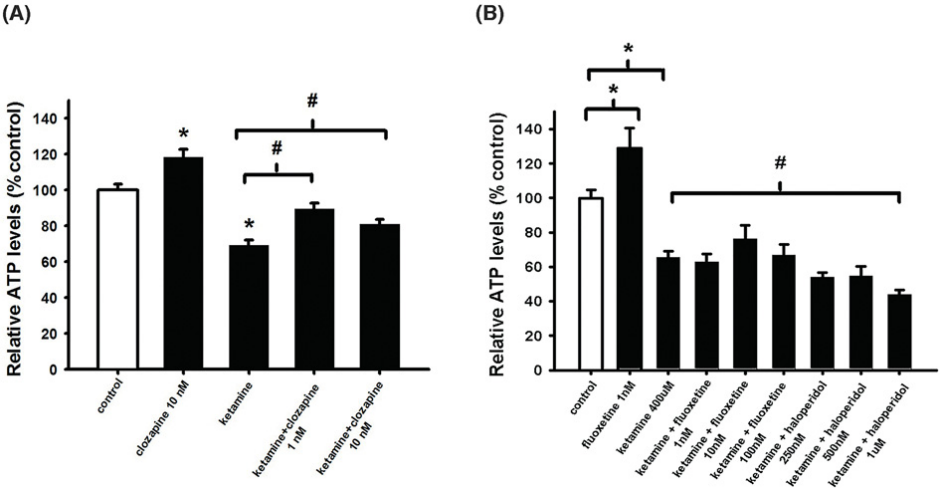
Ketamine-induced cell death counteracted by clozapine, while fluoxetine and haloperidol cannot (**A,B**) NSCs were plated as single cells and treated with 400 μM ketamine and/or treated with 1, 10 nM clozapine or 1, 10, 100 nM of fluoxetine or 250, 500 nM, 1 μM of haloperidol. After 24-h incubation, intracellular ATP levels were measured. Data are shown as mean ± SEM ((A), *n*=23–39; (B), *n*=4–7). Kruskal–Wallis followed by Dunn’s test was used. Differences were considered significant at *P*<0.05. * denotes *P*<0.05 compared with control, ^#^ denotes *P*<0.05 compared with ketamine.

In subsequent experiments, 400 μM of ketamine was selected. As shown in (Figure 2B), both haloperidol and fluoxetine were unable to counteract the ketamine-induced toxic effect on NSCs, while low concentrations of clozapine (1, 10 nM) were able to revert the cytotoxic effect induced by ketamine (Figure 2A).

### Clozapine counteracts ketamine-induced decrease in NSC viability in correlation to decreased apoptosis and autophagy

**Figure 3:**
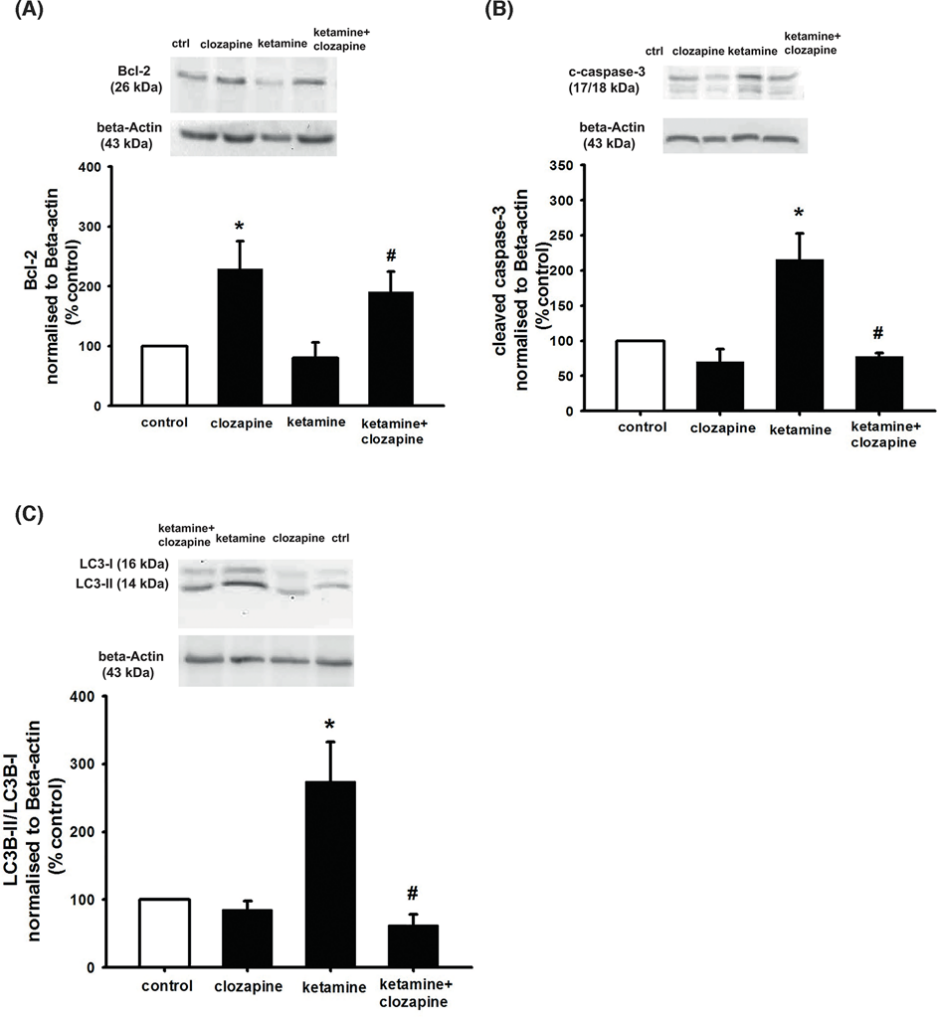
Clozapine counteracts the impaired NSC viability induced by ketamine, in correlation with decreased apoptosis and autophagy NSCs were plated as single cells. Cells were treated with 400 μM ketamine and 10 nM clozapine for 24 h. After 24-h incubation, cells were harvested for Western blot experiments. To obtain quantitative measurements (**A**) Bcl-2 protein levels and (**B**) cleaved caspase 3 (**C**) LC3-II/LC3-I ratio and were normalised against β-actin. Data are shown as mean ± SEM (A), *n*=3–4; (B), *n*=3–4; (C), *n*=3–5. Kruskal–Wallis followed by Dunn’s test was used. Differences were considered significant at *P*<0.05. * denotes *P*<0.05 compared with control, ^#^ denotes *P*<0.05 compared with ketamine.

To study the potential involvement of apoptosis in the counteracting effect of clozapine on NSC viability, we assessed the protein levels of the anti-apoptotic marker Bcl-2 and the pro-apoptotic marker cleaved form of caspase-3 after 24 h of incubation by Western blotting analysis. As shown in Figure 3A clozapine alone increased Bcl-2 expression, while the levels of cleaved form of caspase-3 was unchanged (Figure 3B). Moreover, ketamine-induced a significant decrease in Bcl-2 protein levels and an increase in levels of cleaved form of caspase-3. Finally, co-treatment with clozapine was able to revert these effects (Figure 3A,B). Similar results were obtained with Coomassie normalisation (Supplementary Figure S1A,B).

Autophagy is crucial in maintaining cellular homoeostasis. It constitutes a major protective mechanism that allows cells to survive in response to multiple stressors and helps to defend organisms against degenerative, inflammatory, infectious and neoplastic diseases [55–57]. Importantly, several studies have shown its crucial role in the development and pathology of schizophrenia [58,59]. To study whether ketamine induces autophagy, NSCs were incubated with 400 μM ketamine for 24 h and/or co-treated with 10 nM clozapine whereupon the level of the autophagosome marker LC3-II was assessed. Results in Figure 3C show LC3-II levels to be significantly higher in cells treated with ketamine compared with control, while ketamine combined with clozapine treatment inhibited the ketamine-induced autophagy activity. Clozapine alone did not affect LC3-II activity.

## Discussion

Several studies have reported neurogenesis impairment in schizophrenic patients and in established animal models of schizophrenia induced by non-competitive NMDA-receptor antagonist models of schizophrenia [9,11,12]. Interestingly, in an animal model of schizophrenia, induced by non-competitive NMDA-receptor antagonist PCP, clozapine was shown to counteract a decrease in BrdU-labelling cells of the DG [37] suggesting that clozapine have neurogenesis protective activity. However, phenotypic determination by immunohistochemistry analysis of BrdU-positive cells could not clarify whether BrdU positive cells were indeed NSCs and consequently could not clarify neuroprotective activity of NSCs [37]. Therefore, the aim of the present study was to elucidate whether clozapine has NSC-protective activity and whether this involves an up-regulation of an anti-apoptotic response. To avoid the complexity of an *in vivo* condition, we tested the NSC activity of clozapine in an *in vitro* model. Here we present data indicating that clozapine exerts NSC protective effect, that was associated with an up-regulation of an anti-apoptotic response. We also observed that clozapine may decrease cellular stress, as evident by an attenuated autophagy. Our data are in accordance with previous studies where clozapine has shown cell protective properties in various other cell types *in vitro*, mediated by an anti-apoptotic activity and associated with up-regulation of Bcl-2 activity [38,39,60,61]. However, we cannot conclusively prove at this stage which specific cells are responsible for the reported effects due to the heterogeneity population of NSCs [62]. Therefore, the results of the present study are preliminary and further single cell analysis with appropriate markers will need to be performed to investigate whether the neuroprotective effect of clozapine is linked to a specific subcell population. Overall, our observation of a neuroprotective NSC activity, may thus give further support to the previous *in vivo* study, by Maeda et al. [37], suggesting that clozapine may have a neurogenic-protective activity.

In a previous *in vivo* study by Halim et al. [35] they demonstrated that clozapine may also promote neurogenesis stimulating cell proliferation. However, they were not able to distinguish whether this involved NSC proliferation. In the present study, we used a previously established ATP cell viability assay, specially developed to study cell protection of adult NSCs *in vitro* [24,43,63–65], involving an assay condition omitting cell proliferation. Thus, further studies need to be conducted to clarify whether clozapine also stimulates NSC proliferation, using assay conditions optimised for the analysis of NSC proliferation.

We and others have previously shown that ketamine causes apoptosis [24,66] and up-regulates autophagy [67]. To explore the underlying mechanism of observed NSC protection and anti-apoptotic activity, induced by clozapine, we investigated the effect of clozapine on autophagy. This was conducted by analysing expression levels of LC3-II, a phosphatidylethanolamine modified isoform of the microtubule-associated protein LC3-I, which is generated and translocated to nascent autophagosomes upon macroautophagy induction. Thus, LC3-II is considered a biochemical marker evidence for autophagy in many studies [68–70]. Our results showed that the anti-apoptotic activity of clozapine was associated with attenuated autophagy evident by decreased protein levels of LC3-II. The result was in accordance with previous studies where agents that attenuated ketamine neurotoxicity were associated with decreased apoptosis and autophagy [66,67]. These data suggest that clozapine may inhibit the accumulation of toxic protein aggregates and defective organelles that ketamine introduces to the cells, causing an accumulation of dysfunctional autophagosomes. However, several reports have described that blockage of autophagy in neurons leads to cell death and neurodegeneration in rodents and significant reduction in autophagy in post-mortem hippocampus of schizophrenia patients [58,71,72]. This suggests that autophagy may have a dual role [73], and both be important for the removal of damaged proteins/organelles and promoting cellular injury. Thus, increased or decreased autophagy may be dependent on different injury models and injured cells, although the reasons for this are still unknown [70].

We were also able to detect a cytotoxic activity when NSCs were exposed to ≥500 nM of clozapine. A cytotoxic activity for clozapine is well-established in the literature and has been observed in numerous *in vitro* [68,74,75] and *in vivo* studies [76]. Importantly, a study by Park et al. [68], showed a neurotoxic activity when primary neurons were exposed to ≥10 μM of clozapine *in vitro*, the observed neurotoxic activity was also associated with increased apoptosis and autophagy. Hematopoietic toxicity is the major limiting factor for a general use of clozapine for treating schizophrenia. Patients with schizophrenia treated with clozapine in therapeutic concentrations, have serum levels in the concentration range of 1–2 μM [77,78]. In a study by Nordin et al. [77], the levels of clozapine in cerebrospinal fluid (CSF) of nine treated patients were found to be much lower, in the range of 16–120 nM. Although speculative, if linking these therapeutic concentrations of clozapine to the concentrations of clozapine used in the current study, it may indicate that therapeutic concentrations have the possibility to elicit either cell protective or cytotoxic activity on adult NSCs. This assumption needs to be validated *in vivo* by investigating whether disturbed neurogenesis occurs in the OB and striatum in animal models of schizophrenia. If neurogenesis is disturbed in these areas, it would be of interest to administer clozapine and see whether it can normalise the impaired neurogenesis and neural plasticity in schizophrenia.

## Conclusions

We report that clozapine may have a protective/anti-apoptotic activity on NSCs, alone and against ketamine-induced cytotoxicity, as demonstrated by increased protein expression of the anti-apoptotic marker Bcl-2 and decreased protein expression of the pro-apoptotic marker cleaved form of caspase-3, respectively. Moreover, clozapine’s cell protective activity was associated with decreased protein expression of the autophagasome marker LC3-II. The demonstrated NSC-protective activity, involving up-regulation of an anti-apoptotic response by clozapine, give further support to previous *in vivo* observations indicating a neurogenesis-promoting activity for clozapine. If these data are confirmed *in vivo*, they may encourage an expanded use of clozapine to restore impaired neurogenesis in schizophrenia.

## Supplementary Material

Supplementary Figure S1

## Abbreviations

Bcl-2: B-cell lymphoma 2
bFGF: basic fibroblast growth factor
BrdU: bromodeoxyuridine
CNS: central nervous system
DG: dentate gyrus
EGF: epidermal growth factor
FGA: first-generation
LC3-II: 1A/1B-light chain 3
mtDNA: mitochondrial DNA
mt-ND1: mitochondrial encoded NADH dehydrogenase 1
NMDA: N-methyl-d-aspartate
NS: neurosphere
NSC: neural stem cell
OB: olfactory bulb
PCP: phencyclidine
RPPH1: nuclear-encoded ribonuclease P RNA component H1
SGA: second-generation
SGZ: subgranular zone
SVZ: subventricular zone
