# Supplementary material for: Clozapine protects adult neural stem cells from ketamine-induced cell death in correlation with decreased apoptosis and autophagy
Source: Biosci Rep. 2020 Jan 24;40(1):BSR20193156. doi: 10.1042/BSR20193156 (PMC6981094; doi:10.1042/BSR20193156)
Supplement: Supplementary Figure S1 [file BSR-2019-3156_supp.pdf]

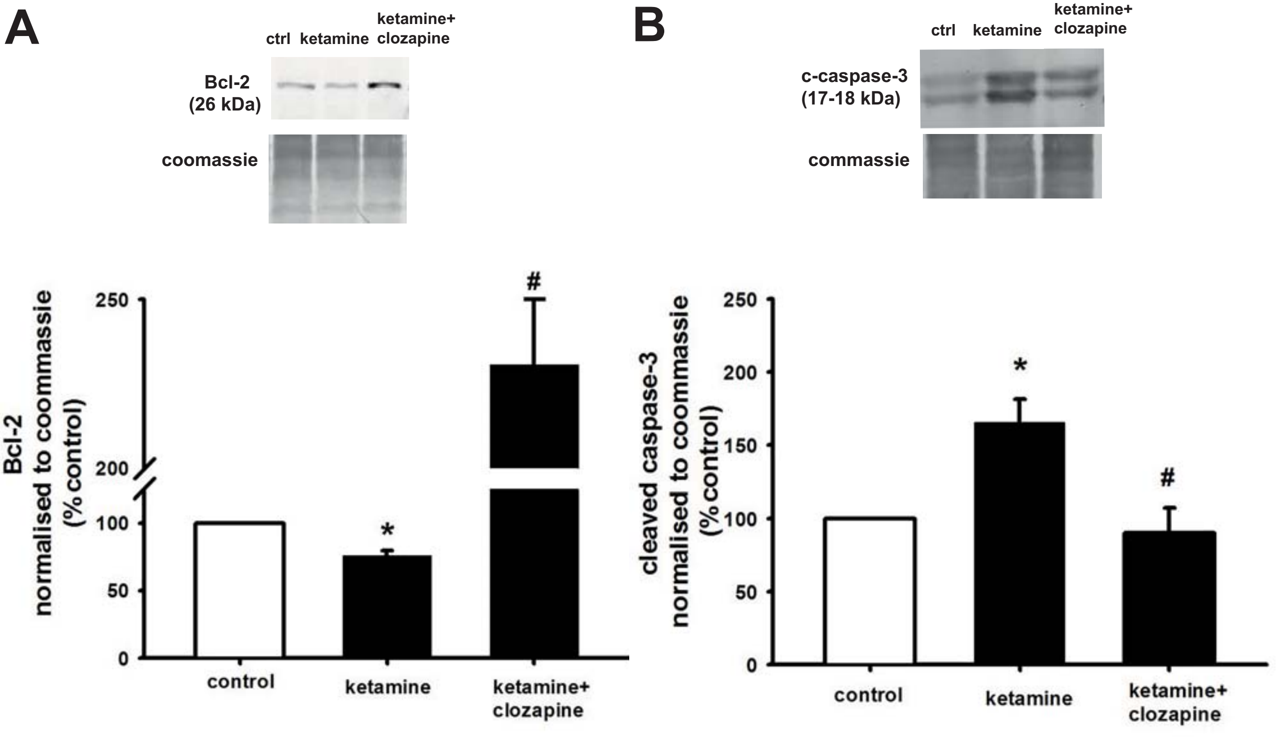
A
ctrl
ketamine
ketamine+
clozapine
Bcl-2
(26 kDa)
coomassie
Bcl-2
normalised to coomassie
(% control)
250
200
100
50
0
#
*
control
ketamine
ketamine+
clozapine
B
ctrl
ketamine
ketamine+
clozapine
c-caspase-3
(17-18 kDa)
commassie
cleaved caspase-3
normalised to coomassie
(%control)
250
200
150
100
50
0
*
#
control
ketamine
ketamine+
clozapine

Suppl Fig.1 Clozapine counteracts ketamine-induced cell death in correlation with decreased apoptosis. NSCs were plated as single cells. Cells were treated with 400 µM ketamine and 10 nM clozapine for 24 hours. After 24 hours incubation cells harvested for Western blot experiments**.** To obtain quantitative measurements **(A)** Bcl-2 protein levels and **(B)** cleaved caspase 3 and were normalized against Coomassie blue. Data are shown as mean ±SEM (A, n=5-6; B, n=4-5). Kruskal-Wallis followed by Dunn's test was used. Differences were considered significant at P <0.05. *denotes P <0.05 compared with control, # denotes P<0.05 compared to ketamine.
